# A study on the prognostic assessment of triple-negative breast cancer using habitat analysis of preoperative DCE-MRI subtraction maps

**DOI:** 10.3389/fonc.2026.1820642

**Published:** 2026-07-03

**Authors:** Yiming Yao, Junfeng Kong, Shanshan Jiang, Yuan Sun, Wanqiu Zhang, Jinding Wei, Sen Xing, Fangsheng Mou, Xinghua Liu, Wenbing Zeng

**Affiliations:** 1 Chongqing University Three Gorges Hospital, Chongqing, China; 2 Bayer Healthcare, Wuhan, China; 3 Chongqing University Fuling Hospital, Chongqing, China

**Keywords:** heterogeneity, magnetic resonance imaging, neoplasm recurrence, prognosis, triple negative breast neoplasms

## Abstract

**Objective:**

Assessment of disease-free survival (DFS) in triple-negative breast cancer (TNBC) using habitat analysis of preoperative DCE-MRI subtraction maps.

**Methods:**

This study retrospectively collected consecutive patients with pathologically confirmed triple-negative breast cancer from January 2019 to January 2025 at our institution for evaluation. Ratio maps of [(peak enhancement-unenhanced period)/unenhanced ROI] for wash-in ratio map, [(peak enhancement-delayed period)/enhanced ROI] for washout ratio map were obtained by DCE subtraction maps. Habitat was calculated based on the clustering of the two subtraction maps ratio maps, and intratumoral heterogeneity (ITH) was calculated based on the proportion of each cluster. Recurrence of TNBC was determined using Kaplan-Meier survival analysis, and multivariable Cox regression analyses were used to identify independent risk factors for TNBC recurrence.

**Result:**

A total of 145 patients with triple-negative breast cancer were finally enrolled, of whom 29 patients experienced recurrence. The results of DCE habitat analysis showed that the habitat ITH was higher in the high-risk recurrence group than that in the low-risk recurrence group (0.59 ± 0.14 vs. 0.42 ± 0.22, *P<* 0.001). In the ROC curve for diagnosis of triple-negative breast cancer recurrence, the AUC for habitat ITH was 0.71 (95% CI: 0.616-0.805, *P* < 0.05). Habitat ITH (HR 1.465, *P* = 0.013) was an independent predictor of poorer DFS in multivariable analysis.

**Conclusion:**

Habitat analysis of preoperative DCE-MRI subtraction maps contributes to the prognostic assessment of TNBC.

## Introduction

Triple-negative breast cancer (TNBC) is an aggressive subtype of breast cancer, accounting for approximately 15–20% of all breast cancer cases, and is associated with poor prognosis and high recurrence rates. Patients with TNBC have a 2.0- to 3.5-fold higher likelihood of recurrence compared to those with other breast cancer subtypes (1). This aggressive behavior is largely attributable to the high intratumoral heterogeneity (ITH) and biological plasticity characteristic of TNBC (2, 3). Notably, high ITH has been associated with worse distant metastasis–free survival in invasive breast cancer (4). Therefore, precise preoperative quantification of tumor heterogeneity is of critical clinical significance for predicting treatment response, guiding individualized therapeutic decisions, and stratifying prognostic risk in TNBC. Currently, assessment of breast ITH relies primarily on tissue sampling and pathological evaluation (5). However, traditional histopathological quantification has inherent limitations, including invasiveness, sampling error, and poor reproducibility. Consequently, there is an urgent need to develop a noninvasive, reproducible imaging method capable of comprehensively assessing tumor heterogeneity and to establish effective imaging biomarkers associated with the risk of TNBC recurrence.

Magnetic resonance imaging, particularly dynamic contrast-enhanced MRI (DCE-MRI), has become an essential tool in breast cancer assessment due to its ability to reflect tumor blood perfusion and microvascular permeability. However, conventional DCE-MRI analysis often relies on whole-tumor average parameters (e.g., K^trans^), an approach that tends to obscure the complex spatial heterogeneity within the tumor, where regions of reduced blood flow (e.g., vascular normalization zones) and increased blood flow (e.g., areas of sustained angiogenesis) may coexist (6). Relying solely on traditional parametric maps is insufficient to fully characterize the underlying biological differences within tumors, thereby limiting the accurate assessment of aggressive behavior in TNBC.

Habitat analysis, an emerging imaging methodology, offers a novel approach for noninvasive assessment of tumor heterogeneity by delineating subregions with distinct biological functions within the tumor. It has demonstrated promising applications in studies predicting pathological response to breast cancer treatment (7) and assessing HER2 status (8). Habitat subtraction mapping, in particular, is a method that fully leverages the dynamic contrast-enhanced characteristics of DCE sequences to characterize spatial heterogeneity, which encompasses the heterogeneity of the tumor bulk, its subregions, and the surrounding area (9, 10). The analysis of DCE subtraction maps using semi-quantitative or quantitative parameters enables the evaluation of regional intratumoral heterogeneity, directly reflecting biological information related to proliferative activity, cellular characteristics, or microvascular properties (9, 11).

Although previous studies have explored habitat analysis, certain limitations still exist. A limitation of previous MR habitat analysis lies in the subregions automatically segmented via unsupervised clustering or deep learning (12), whose biological interpretation may be ambiguous. This is partly because radiomics is primarily focused on data-driven feature extraction, and many texture features lack a clear biological basis (13–15), making their implications difficult to interpret. Furthermore, due to the high spatial and temporal heterogeneity of angiogenesis in malignant tumors, it is challenging to fully characterize tumor biology using DCE images alone. Spatial heterogeneity plays a critical role in driving cancer progression and promoting resistance to systemic therapies (16). Consequently, we propose that the hidden information within DCE-MRI sequences, particularly DCE subtraction maps, has not been fully explored or utilized, and that such maps offer greater biological interpretability. To date, few studies have systematically investigated the principle of habitat generation from subtraction maps in TNBC patients, nor has the association between subtraction map-derived habitat features and prognosis in TNBC patients been established.

Based on this, we hypothesize that habitat analysis derived from preoperative DCE-MRI subtraction maps can noninvasively and quantitatively capture the intratumoral heterogeneity of triple-negative breast cancer, and that these heterogeneity features are closely associated with patient prognosis. Therefore, this study aimed to perform habitat analysis using preoperative DCE-MRI subtraction maps to investigate the value of quantitative habitat parameters in assessing tumor heterogeneity in TNBC, thereby providing a novel imaging basis for precise risk stratification and prognostic evaluation in TNBC.

## Methods

### Patients

**Figure 1:**
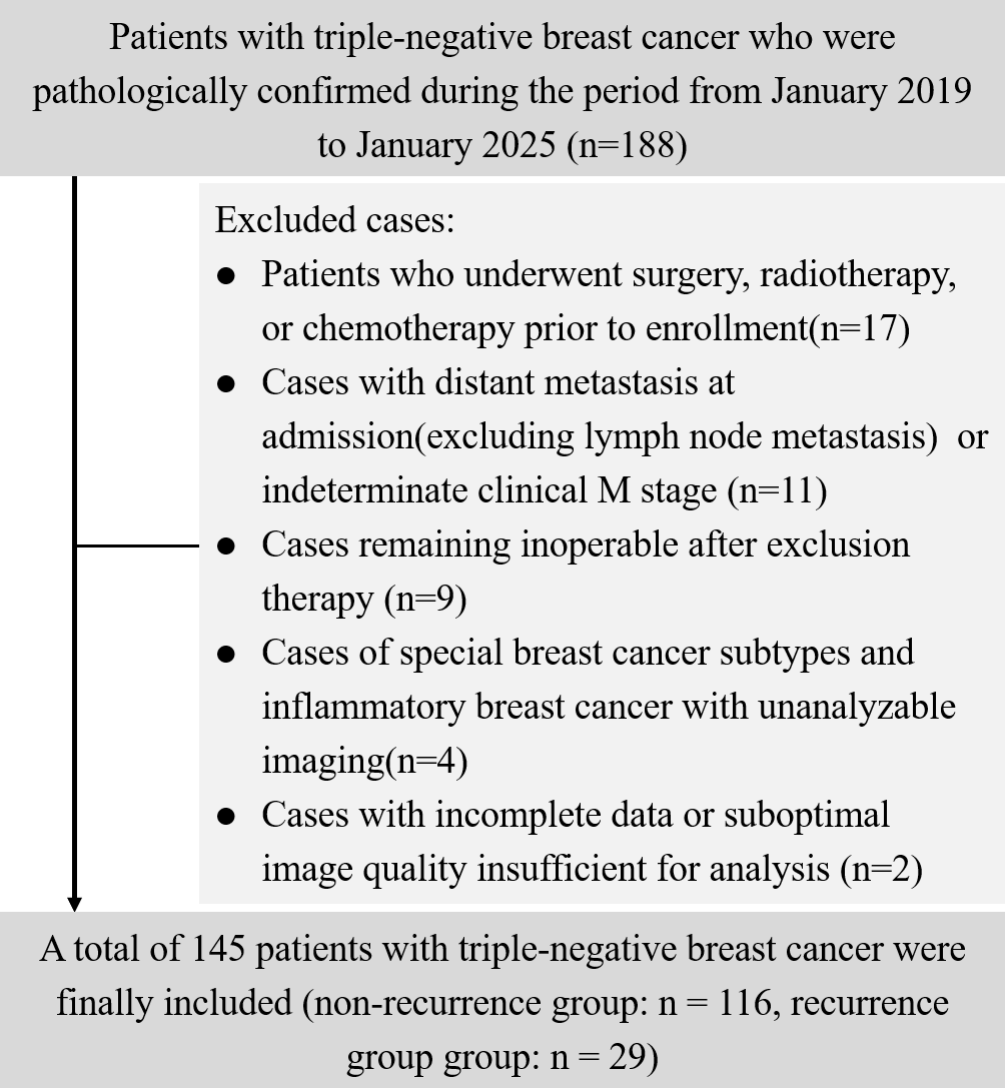
Flowchart of the study population.

This retrospective study was approved by our institutional ethics committee and informed consent was waived. This study consecutively collected 188 patients with pathologically confirmed triple-negative breast cancer(TNBC) from our institution between January 2019 and January 2025 for evaluation. TNBC was defined according to ASCO/CAP guidelines as: ER<1%, PR<1%, and HER2 negative (IHC 0 or 1+; or IHC 2+ with negative FISH). Inclusion criteria: i) age ≥ 18 years, female, with or without menopause; ii) pathologically confirmed triple-negative breast cancer by puncture or surgical biopsy; and iii) completion of the first MRI before puncture. Exclusion criteria: ① patients who had received surgery, radiotherapy and chemotherapy treatment before enrollment; ② cases with distant metastasis (but not lymphatic metastasis) or clinical M-staging that could not be determined at the time of enrollment; ③ exclude cases that still could not be operated after treatment; ④ exclude special types of breast cancer as well as inflammatory breast cancers that could not be analyzed by the images; and ⑤ exclude patients with incomplete and imperfect information or poor image quality that was insufficient to satisfy the analyses. **Figure 1** illustrates the flow chart of the study population.

The collected information included patients’ clinical and demographic characteristics (age, menopausal status, family history, clinical stage, treatment status, and recurrence status), pathological information (ER/PR/HER2 status, Ki-67 index, histological grade, and treatment response), and baseline MRI data (morphological features, DCE data, and related derived data). Recurrence status was retrospectively assessed using the electronic medical record system and the PACS system.

The outcome metric for our study was disease-free survival (DFS) definition (17): it is the time from surgery or neoadjuvant therapy followed by surgery to disease recurrence or death, as well as the time to the last known evidence of absence of disease or the most recent follow-up. Median DFS: the time to disease recurrence or death in 50% of patients. Definition of recurrence: a situation in which TNBC is treated to achieve a state of complete clinical remission and the tumor reappears as a tumor lesion at the primary site or elsewhere in the body (local recurrence, recurrence in regional lymph nodes, or distant metastasis). recurrence within 5 years was defined as the high-risk group for recurrence; otherwise, the group was defined as the low-risk group for recurrence. The last follow-up was on 01/04/2025.

### MRI image parameters

The MRI scanner used in our study was the Vireo 3.0T scanner (Verio^®^, Siemens Healthcare), equipped with an eight-channel breast-specific coil. Patients were positioned prone, with both breasts naturally hanging into the coil opening. Scanning protocol: conventional non-contrast scanning followed by DCE scanning. Scanning parameters: axial T1-weighted imaging (T1WI), TE 2.45 ms, TR 6.04 ms, FOV 330 × 330 mm, slice thickness 1.5 mm, no gap; axial fat-suppressed T2-weighted imaging (T2WI) non-contrast scanning, TE 61 ms, TR 4300 ms, FOV 330 × 330 mm, slice thickness 3.5 mm, no gap; diffusion-weighted imaging, b-value 0–1000 s/mm², FOV 180×320 mm, slice thickness 3.5 mm; DCE used a three-dimensional fast low-angle fat-suppressed T1WI sequence, TE 1.71 ms, TR 4.77 ms, FOV 330×330 mm, slice thickness 1.5 mm, no gap. Gd-DTPA (Omniscan, GE Healthcare) at a dose of 0.1 mmol/kg was injected via the antecubital vein using a high-pressure syringe at a flow rate of 2.5 ml/s. Following injection, 20 ml of saline was injected at the same flow rate. Pre-contrast images were acquired prior to dynamic enhancement, followed by six consecutive dynamic enhancement scans, each lasting one minute (60 seconds). The scanning range extended from the axilla to the diaphragm, with the primary field of view including both breasts, the anterior chest wall, and the axilla. After scanning, the images were uploaded to the Siemens workstation, and conventional time-signal intensity curve (TIC) processing was performed on the tumor lesions. The curves were classified into three types: Type I (ascending), Type II (plateau), and Type III (washout).

### Analysis of basic characteristics of DCE-MRI images

Two radiologists (A and B, with 7 and 3 years of experience, respectively) performed image analysis without knowledge of the pathological results. The main indicators analyzed were tumor morphology, TIC type, background parenchymal enhancement (BPE), edge enhancement, necrosis, peritumoral edema, and multifocality.

### Habitat analysis

First, image motion correction was performed with translation displacement less than 1 mm and rotational displacement less than 5°considered satisfactory. The non-enhanced phase, peak enhancement phase, and delayed phase of all cases were imported into ITK-SNAP (version 3.8.0, http://www.itksnap.org/pmwiki/pmwiki.php?n=Main.HomePage) for image ROI delineation. If there was a single lesion, layer-by-layer ROI delineation was performed of the lesion, avoiding areas with obvious necrosis, cystic changes, or hemorrhage. If there were multiple lesions, the largest lesion was selected for the above steps. The preprocessing process is to align the DCE image and then resample the enhanced peak period and delayed period to the unenhanced period space, so that the origin, spacing, direction and size of the 3 maps are consistent. Then the three images were resampled to 1 mm × 1 mm × 1 mm.

Ratio maps were obtained from the silhouette analysis: enhanced-unenhanced ratio map [(enhanced peak image - unenhanced image)/unenhanced ROI] and enhanced-delayed ratio map [(enhanced peak image - delayed image)/enhanced ROI]. Habitats were calculated by clustering these two silhouette ratio maps, and ITH was computed based on the proportion of each cluster. Silhouette image and habitat analysis utilized Python v3.9.13 and scikit-learn v1.6.1 machine learning package. The unenhanced phase refers to the images at the start of DCE without contrast agent injection. The enhanced peak phase refers to the images at 90 seconds after contrast agent injection (Phase 2 in this study). The delayed phase refers to the images at 360 seconds (Phase 6 in this study).

**Figure 2:**
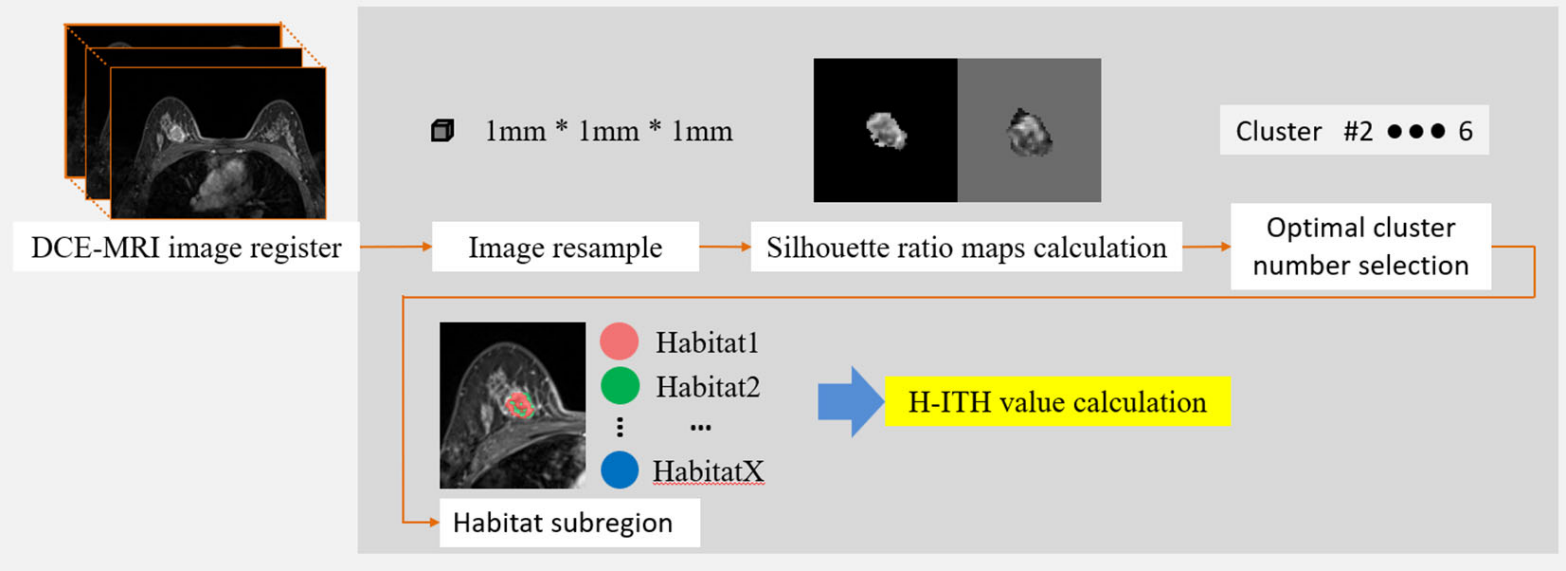
Flowchart of the habitat analysis. DCE-MRI, dynamic contrast-enhanced MRI; H-ITH, habitat intratumoral heterogeneity.

The specific steps for habitat analysis were as follows: ① Silhouette ratio maps calculation: enhanced-unenhanced ratio and enhanced-delayed ratio maps defined above were calculated as features. ② Clustering: the features were Z-score standardized and clustered using k-means, and the silhouette score (parameter settings: sample size = 5000) was used to select the optimal number of clusters from 2 to 6. ③ ITH refers to a measure of heterogeneity calculated from the proportion of voxels in habitat subregions. H-ITH calculation: the proportion of each habitat was calculated and the final $\text{H-ITH}=-\overset{\text{k}}{\underset{\text{i}=1}{\sum }}p_{i}\text{log}\left(p_{i}\right)$ (0 ≤ *p_i_* ≤ 1, where log refers to the natural logarithm (base e), *p_i_* is the proportion of the i-th cluster, k is the number of clusters, and ITH ranges from 0 to log(k), with higher values indicating greater heterogeneity). **Figure 2** shows the processing flowchart.

### Statistical analysis

Statistical analyses were performed using SPSS 25.0 software. Continuous numerical variables were compared using Student’s t-test and the results were expressed as mean ± standard deviation $\left(\bar{x}\pm s\right)$. Categorical variables were compared using chi-square test or Fisher’s exact test. Kappa coefficients were used to assess inter-observer agreement between the two observers: 0.81-1.00 for almost perfect agreement; 0.61-0.80 for substantial agreement; 0.41-0.60 for moderate agreement; 0.21-0.40 for mild agreement; and 0.20 and below for weak agreement (18). The final results were reached by consensus.

Receiver operating characteristic (ROC) curves were plotted to analyze the diagnostic value of the parameters. The area under the curve (AUC), sensitivity, specificity, positive predictive value (PPV), and negative predictive value (NPV) were calculated, and the optimal cutoff value for ITH was determined using the Youden index (sensitivity + specificity – 1). Kaplan-Meier survival analysis, univariable and multivariable Cox regression analyses were used to determine the association between habitat analysis and TNBC recurrence and to obtain the risk ratio (HR) and its 95% confidence interval (CI). The variance inflation factor (VIF) was used to assess multicollinearity among the variables. A VIF > 5 was considered to indicate significant multicollinearity. The final results were reached by consensus. *P*-value<.05 was considered statistically significant.

## Result

### Baseline characteristics

Initially, 188 patients with triple-negative breast cancer were enrolled. Exclusions included 3 cases who had undergone chemotherapy prior to presentation, 14 cases who had undergone surgery or minimally invasive procedures prior to consultation, and 11 cases presenting with distant metastasis (excluding lymph node metastasis) or indeterminate clinical M stage at admission. We further excluded 9 cases remaining inoperable post-treatment, as well as special breast cancer subtypes and inflammatory breast cancer with unanalyzable imaging (1 myxocarcinoma, 1 adenoid cystic carcinoma, 2 inflammatory breast cancers). and 2 cases with incomplete or inadequate data or poor image quality. Ultimately, 145 patients with TNBC were included, among whom 29 patients experienced recurrence, accounting for approximately 20%. The median time to recurrence was 19.6 months (range, 2.9–58.0 months), and the median follow-up time was 30.6 months (range, 0.2–75.4 months). In the high-risk recurrence group, there were 6 cases of local recurrence, 20 cases of regional lymph node recurrence or distant metastasis, and 3 cases of both local recurrence and distant metastasis.

Among the general characteristics, patients in the high-risk recurrence group were younger than those in the low-risk recurrence group (47.72 ± 12.72 vs. 53.23 ± 8.42, *P* = 0.034) (**Table 1**). The differences between the DCE-MRI images of the two groups were statistically significant in terms of BPE, rim enhancement signs, and peritumoral oedema (*P*-values of.03,.001, and.005, respectively). In addition, there was a very good agreement between the two observers (Observer A and Observer B) in terms of the basic signs of the images, with Kappa values > 0.80 (**Table 2**).

**Table 1:** The general clinical characteristics of the patients.

Characteristic	Low-risk recurrence group (*n* = 116)	High-risk recurrence group (*n* = 29)	t/χ²	*P-*value
Age (years)	53.23 ± 8.42	47.72 ± 12.72	2.21	0.034
Menopause	76 (65.5%)	12 (41.37%)	5.66	0.017
There is a family history	5 (4.31%)	0 (0)	–	0.058
Tumor length (cm)	2.96 ± 1.35	3.79 ± 2.00	-2.10	0.042
Ki-67 (>20%)	108 (93.1%)	27 (93.1%)	–	1
Treatment method				
Chemotherapy	41	15	2.62	0.105
Surgery	75	14		
Clinical T stage				
T1-3	10	22	–	0.046
T4	106	7		
Clinical N stage				
N0	64	10	3.97	0.046
N1 or above	52	19		
Histological grading				
Grade One to Two	37	7	1.07	0.3
Grade Three	47	15		
Unknown	32	7		

- Unless otherwise indicated, data in parentheses are percentages. Mean data are ± SDs.

**Table 2:** Basic features of images and inter-observer comparisons.

Characteristic	Observer A		χ²	*P*-value	Observer B		χ²	*P-*value	Kappa value
	Low-risk recurrence group (*n* = 116)	High-risk recurrence group (*n* = 29)			Low-risk recurrence group (*n* = 116)	High-risk recurrence group (*n* = 29)			
Lesion morphology									
Quasi-circular	76	14	2.92	0.087	74	13	3.47	0.062	0.87*
Irregular	40	15			42	16
TIC type									
Type I or II	60	12	0.99	0.319	65	12	2.00	0.157	0.9*
Type III	56	17			51	17
BPE									
Very little – mild (≤50%)	104	21	–	0.03	103	23	1.83	0.176	0.85*
Moderate – severe (>50%)	12	8			13	6
Marginal enhancement sign	53 (45.68%)	23 (79.31%)	10.51	0.001	63 (54.31%)	23 (79.31%)	6	0.014	0.86*
Obvious necrosis	64 (55.17%)	21 (72.41%)	2.84	0.092	65 (56.03%)	23 (79.31%)	5.26	0.022	0.87*
Peritumoral edema	50 (43.1%)	21 (72.41%)	7.97	0.005	47 (40.51%)	19 (65.51%)	5.84	0.016	0.87*
Multiple lesions	18 (15.51%)	4 (13.79%)	–	1	18 (15.51%)	5 (17.24%)	0.05	0.82	0.81*

- Unless otherwise indicated, data in parentheses are percentages. TIC, time-signal intensity curve; BPE, background parenchymal enhancement.

*P values are all<.001. U values for lesion morphology, TIC type, BPE, marginal enhancement sign, obvious necrosis, peritumoral edema and multiple lesions were 10.48, 10.9, 10.26, 10.46, 10.49, 10.56, 9.82 respectively.

### Results of DCE habitat analysis

**Figure 3:**
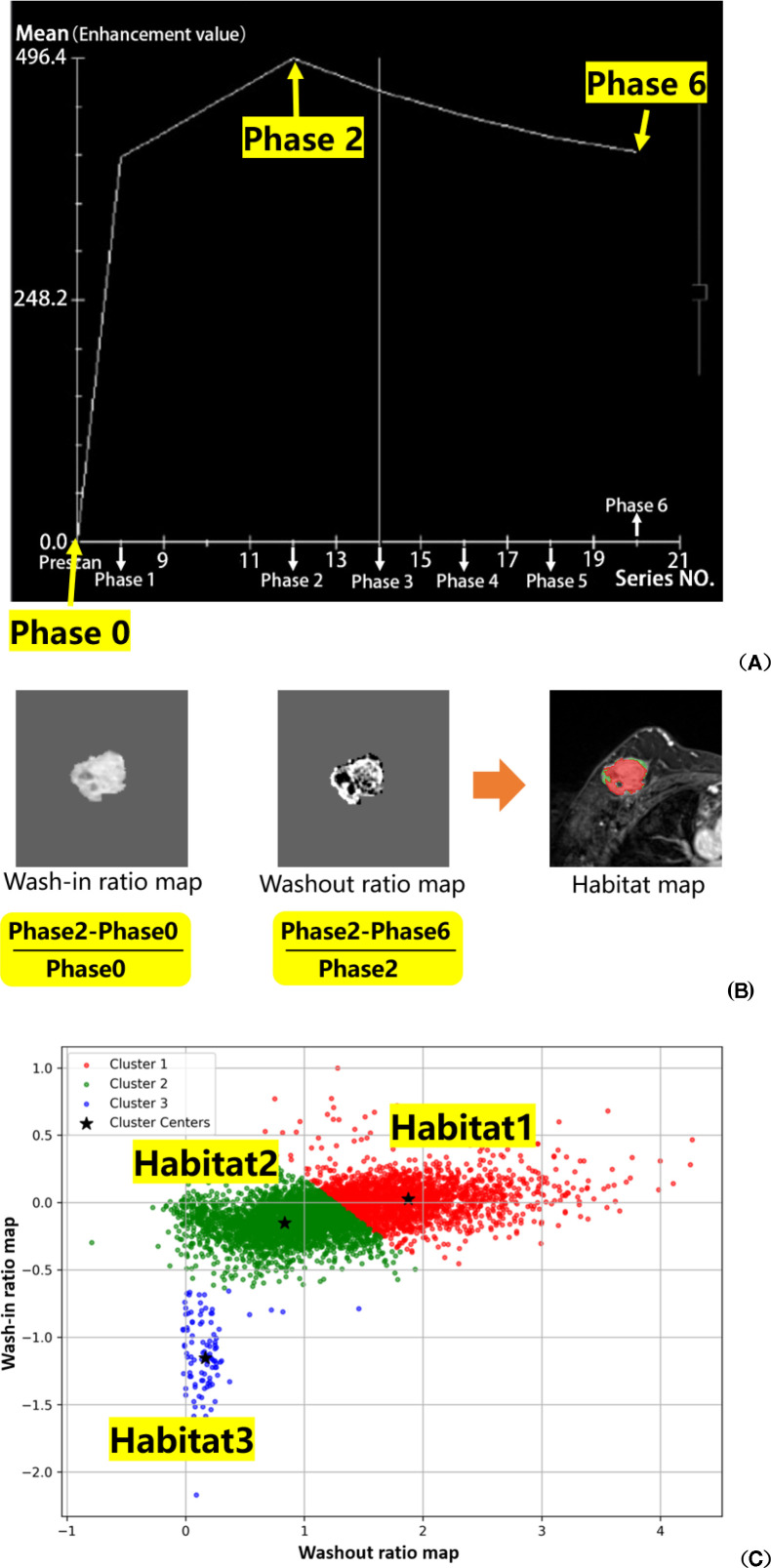
Principle of habitat generation from subtraction maps. **(A)** Dynamic curves from DCE scan sequences, showing the three phases required in this study. **(B)** Illustration showing that the habitat map is generated from the wash-in ratio map and the washout ratio map. **(C)** Graph of feature space clustering results, ★ shows the clustering center. Cluster 1 (habitat1), Cluster 2 (habitat2), and Cluster 3 (habitat3) represent the “early rapid enhancement and rapid washout” features of high, medium, and low specific aggressive meanings, respectively.

The silhouette scores were: k=2: 0.4068, k=3: 0.4407, k=4: 0.3420, k=5: 0.3283, k=6: 0.3181. The highest score at k=3 confirmed that three habitats were optimal. The results showed that the habitat ITH was higher in the high-risk recurrence group than in the low-risk recurrence group (*P* < 0.001). the differences in habitat1 ratio, habitat2 ratio, and habitat3 ratio were not statistically significant (*P* > 0.05) (**Figure 3**; **Table 3**).

**Table 3:** The results of DCE habitat analysis.

Variables	Low-risk recurrence group (*n* = 116)	High-risk recurrence group (*n* = 29)	t	*P-*value
Habitat ITH	0.42 ± 0.22	0.59 ± 0.14	-4.90	<0.001
Habitat1 ratio	0.47 ± 0.33	0.54 ± 0.22	-1.41	0.162
Habitat2 ratio	0.52 ± 0.33	0.45 ± 0.22	1.43	0.156
Habitat3 ratio	0.00 ± 0.03	0.00 ± 0.01	-0.25	0.802

- Unless otherwise indicated, data in parentheses are percentages. Unless otherwise noted, data are mean ± SD. ITH, intratumoral heterogeneity.

### ROC curve and Kaplan-Meier survival analysis curve

In the survival analysis of this study, the median DFS had not been reached. the proportion of cumulative survival at 1, 3 and 5 years was 93.4%, 79.0% and 66.5%, respectively.

**Figure 4:**
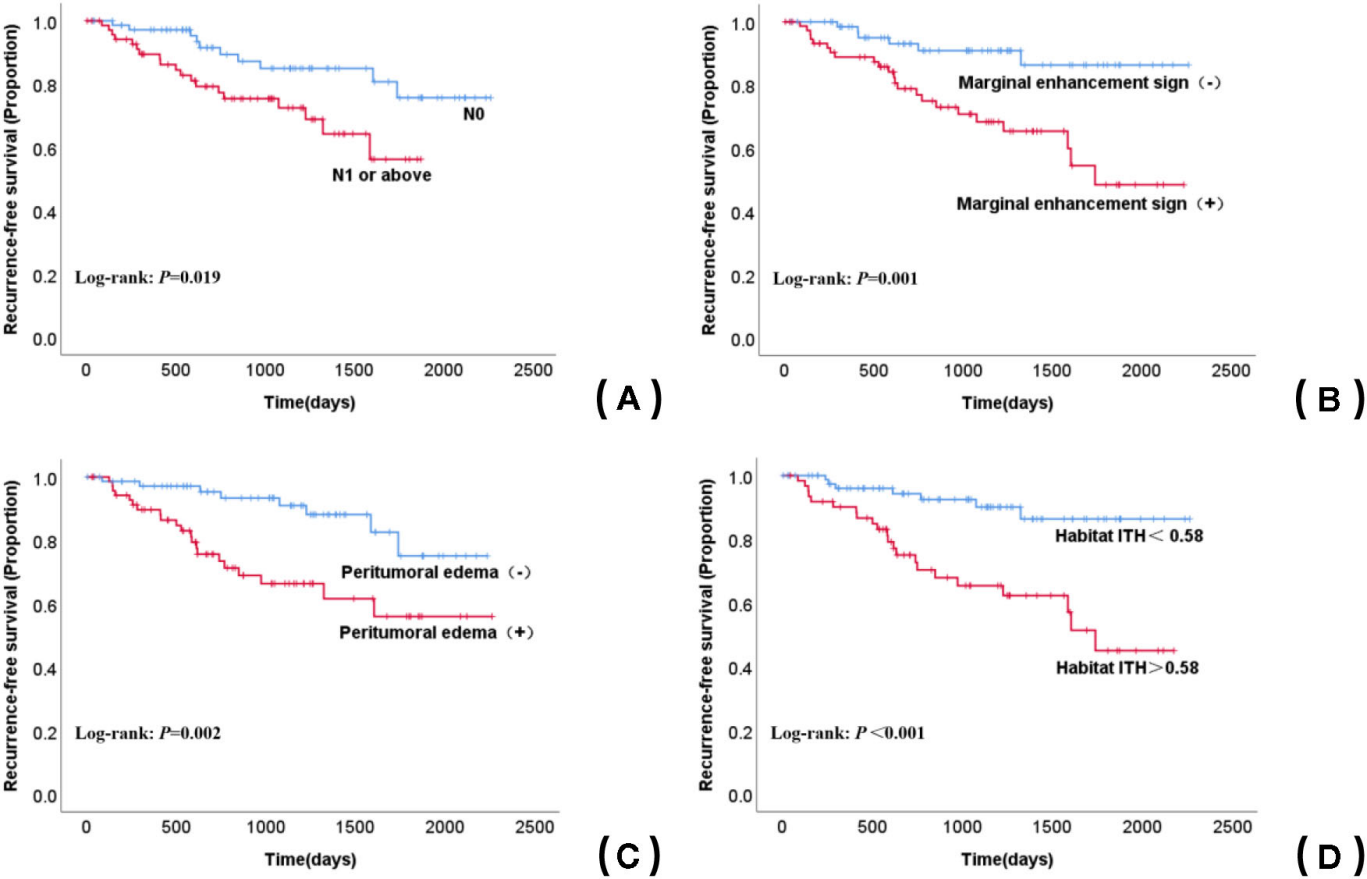
Kaplan-Meier survival analysis curves based on clinical and imaging characteristics of triple-negative breast cancer patients. **(A–D)** It can be seen that the Kaplan-Meier survival analysis curves were significantly lower when the clinical N stage was at N1 or above, the marginal enhancement sign and the peritumoral edema were positive, and the habitat ITH was > 0.58 (*P* < 0.05). ITH=intratumoral heterogeneity.

**Figure 5:**
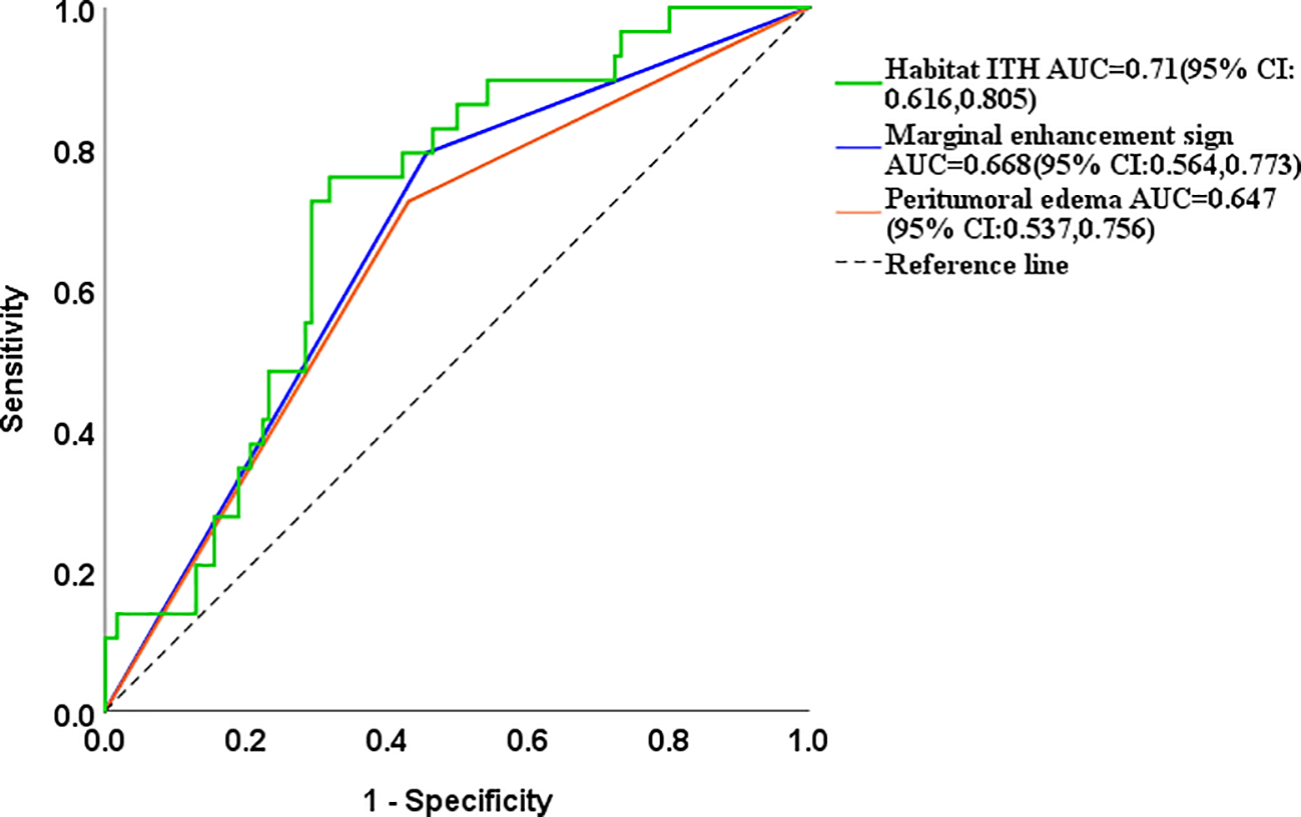
ROC chart for diagnosing recurrence in patients with triple-negative breast cancer. ITH, intratumoral heterogeneity; AUC, area under curve.

In the ROC curves for diagnosing recurrence of TNBC, the AUC values for H-ITH, marginal enhancement sign, and peritumoral edema were 0.71, 0.668, and 0.647, respectively (all *P* values< 0.05). BPE was not statistically significant for the recurrence of TNBC (**Table 4**). When the clinical N stage was N1 or higher, the marginal enhancement sign was positive, peritumoral edema was positive, and ITH > 0.58, the Kaplan-Meier survival analysis curve showed a significant decrease (*P* < 0.05) (**Figures 4**, **5**).

**Table 4:** ROC curve analysis of imaging features.

	AUC(95%CI)	Sensitivity	Specificity	PPV	NPV	*P-*value
Habitat ITH	0.71(0.616-0.805)	0.759	0.681	0.372	0.918	<0.001
BPE	0.586(0.463-0.71)	0.276	0.897	0.275	0.896	0.152
Marginal enhancement sign	0.668(0.564-0.773)	0.793	0.543	0.793	0.543	0.005
Peritumoral edema	0.647(0.537-0.756)	0.724	0.569	0.724	0.568	0.015

- ROC, receiver operating characteristic; ITH, intratumoral heterogeneity; BPE, background parenchymal enhancement; AUC, area under curve; CI, confidence interval; PPV, positive predictive value; NPV, negative predictive value.

### Univariable and multivariable Cox regression

In univariable analysis, age, clinical T stage, clinical N stage, treatment method, tumor length, marginal enhancement sign, peritumoral edema, and ITH were associated with worse triple-negative breast cancer DFS correlation.

The multicollinearity diagnosis revealed no significant multicollinearity among the variables. The VIF values for age, clinical T stage, clinical N stage, treatment method, tumor length, marginal enhancement sign, peritumoral edema, and ITH were 1.066, 1.329, 1.502, 1.754, 1.756, 1.054, 1.158, and 1.085, respectively, all well below the threshold of 5.

**Figure 6:**
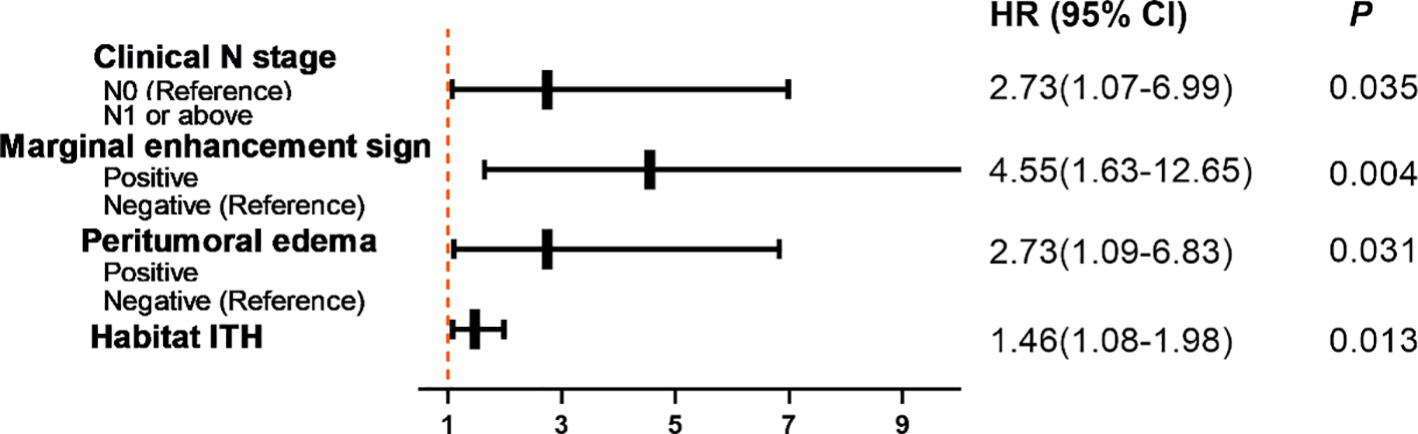
Multivariable Cox-regression analysis for disease-free survival (DFS) between high-risk recurrence group and low-risk recurrence group. ITH, intratumoral heterogeneity. (The smallest single change of habitat ITH is 0.1).

In multivariable analysis, clinical N stage (N0 vs. N1 or above, HR 2.739, *P* = 0.035), marginal enhancement sign (HR 4.551, *P* = 0.004), peritumoral edema (HR 2.739, *P* = 0.031), ITH (HR 1.465, *P* = 0.013) were independent predictors of worse DFS in triple-negative breast cancer (**Figure 6**; **Table 5**).

**Table 5:** Univariable and multivariable Cox analysis of disease-free survival in patients with triple-negative breast cancer.

Variables	Univariable			Multivariable		
	HR	95%CI	*P-*value	HR	95%CI	*P-*value
Age	0.951	0.951-0.99	0.013	0.966	0.93-1.003	0.068
Menopause	0.534	0.255-1.119	0.097			
Clinical T stage	2.609	1.111-6.131	0.028	0.527	0.165-1.681	0.279
Clinical N stage	2.463	1.133-5.355	0.023	2.739	1.073-6.991	0.035
Histological grading	1.087	0.44-2.685	0.857			
Treatment method	2.085	1.004-4.332	0.049	1.21	0.446-3.285	0.708
Tumor length	1.286	1.056-1.567	0.012	1.184	0.899-1.558	0.229
Lesion morphology	1.756	0.847-3.639	0.13			
TIC type	1.302	0.62-2.736	0.485			
BPE	2.147	0.95-4.851	0.066			
Marginal enhancement sign	3.951	1.608-9.713	0.003	4.551	1.636-12.655	0.004
Obvious necrosis	2.14	0.947-4.835	0.067			
Peritumoral edema	3.353	1.481-7.589	0.004	2.739	1.098-6.831	0.031
Multiple lesions	0.831	0.289-2.391	0.731			
Habitat ITH^a^	58.81	3.858-896.357	0.003	1.465	1.083-1.983	0.013
Habitat1 ratio	1.522	0.438-5.285	0.509			
Habitat2 ratio	0.642	0.182-2.259	0.49			
Habitat3 ratio	3.001	0-37823.435	0.82			

- HR=, CI, confidence interval; TIC, time-signal intensity curve; BPE, background parenchymal enhancement; ITH, intratumoral heterogeneity.

^a^ The smallest single change is 0.1.

## Discussion

In this study, we performed noninvasive quantitative assessment of intratumoral heterogeneity in triple-negative breast cancer (TNBC) using habitat analysis of DCE-MRI subtraction maps. Our findings demonstrate that habitat heterogeneity (HR 1.465, *P* = 0.013) is an independent risk factor for recurrence in TNBC and is significantly associated with disease-free survival. Furthermore, we identified three distinct intratumoral habitat subregions—habitat1 ratio, habitat2 ratio, and habitat3 ratio—which exhibited “early rapid enhancement and rapid washout” characteristics corresponding to high, intermediate, and low degrees of aggressive biological behavior, respectively. This suggests that DCE subtraction maps are biomarkers that carry biological information and are more biologically interpretable than habitat subregions extracted from data and features alone.

Recently, Li et al. (12) used phase II DCE-MRI images to construct habitats that could quantify ITH. they divided the tumors into three sub-regions with similar characteristics. However, their study did not provide a clear biological explanation of the meaning of habitat subregions. Chen et al. (19) human study each tumor was divided into three sub-regions (1): high metabolic region, junction region and edge region. However, we believe that the meanings of features extracted by relevant software (e.g., Pyradiomics Python package) do not directly reflect the high metabolic situation, but rather the high vascular distribution. One of the more interpretable habitat subregions is the animal experiments of Kazerouni et al. (20) and Syed et al. (7), who used the kinetic parameters K [trans], KEP, VE values, and Apparent Diffusion Coefficient (ADC) values to generate three habitat subregions: high vascularity-hypercellularity (HV-HC), low vascularity-hypercellularity (LV-HC), low vascularity-low cellularity (LV- LC) and correlated with histology. However, the low resolution of ADC maps and the problem of image artefacts seriously hinder their general use in habitat analysis. Another interpretable habitat subregion is the habitat subregion based on DCE subtraction maps used in this study. Cho et al. (9) identified five perfusion-based habitats using DCE perfusion heterogeneity analysis and constructed a habitat risk score (HRS), which was an independent predictor of worse DFS in the validation cohort (HR = 3.274, 95% CI: 1.378–7.782; *P* = 0.014). In our study, we also found that habitat ITH was an independent predictor of worse DFS (HR = 1.465, *P* = 0.013). Both our study and Cho et al.’s demonstrate the value of ITH in predicting prognosis in breast cancer. Although direct comparison is limited by differences in study populations (all breast cancer subtypes vs. TNBC-only), sample sizes, and analytical pipelines, our study differs from Cho et al. in two key aspects: (i) fewer habitats reduce the complexity of ITH calculation and the risk of model overfitting, which is particularly important for studies with relatively modest sample sizes; and (ii) our method specifically targets TNBC, an aggressive subtype with distinct biological behavior.

Further, in this study, habitat1 ratio actually represents early rapid intensification and rapid washout (**Figure 3**). In the high-risk recurrence group of triple-negative breast cancer was the highest habitat1 ratio (percentage of 0.54 ± 0.22), while in the low-risk recurrence group was the highest habitat2 ratio (percentage of 0.52 ± 0.33), due to the fact that more aggressive tumor subregions may drive disease progression and extend into recurrent tumors (21). Habitat ITH is essentially a concept belonging to Shannon entropy (15, 22), i.e. describing the degree of disorder within a system to quantitatively measure ITH, providing an adjunct to non-invasive analysis of intratumoral signaling complexity. In this study, the habitat ITH was significantly higher in the high-risk recurrence group than in the low-risk recurrence group (*P* < 0.001), suggesting that TBNC with a propensity for recurrence exhibit greater intratumoral heterogeneity. This finding is consistent with prior MRI-based habitat analyses demonstrating that intratumoral heterogeneity is closely associated with poor prognosis in breast cancer (9, 21). In addition, the perfused habitat maps in this study take full advantage of the hemodynamic characteristics of the tumor as it dynamically enhances, and Mahrooghy et al. (23) claimed to have suggested the use of entropy and intensity variance as a scheme for the allocation of tumors to heterogeneity. Since on DCE-MRI images, tumor pixels carry bioinformative features in space and time, and this information is aptly represented by heterogeneity.

Currently, there is no clear and directly usable reference value of habitat ITH for clinical decision-making on TNBC recurrence judgement. Therefore, the survival analysis in this study provides a referable ITH threshold, i.e., higher habitat ITH (>0.58) is associated with poorer DFS (*P* < 0.05). The first reason is the inadequacy of treatment; highly heterogeneous tumors are difficult to completely remove tumor heterogeneity by neoadjuvant therapy or even surgery (24), and the heterogeneity of the residual tumor leads to incomplete treatment, which results in an increased risk of local recurrence or distant metastasis after surgery (shorter DFS). Secondly, there are drug-resistant subpopulations of TNBC, especially those associated with BRCA1/2 mutations, which are often associated with ‘homologous recombination defects’, resulting in cells that are prone to mutations, and the greater the number of mutations, the greater the likelihood of generating different subclones within the tumor (25), and the greater the heterogeneity, which is the main reason why TNBC are resistant to standard chemotherapy. one of the reasons for TNBC resistance to standard chemotherapy. In even if there is an initial response to chemotherapy, the resistant cell subpopulations are able to survive, eventually leading to tumor recurrence. Finally, the high heterogeneity at the genetic level (i.e., at the gene level) determines the association with poor clinical prognosis, immune escape, and treatment resistance (26). In addition, the Kaplan-Meier survival analysis curves of triple-negative breast cancer patients were significantly lower (*P* < 0.05) when the clinical N stage was at N1 and above, when the marginal enhancement sign was positive, and when the peritumoral edema was positive, in agreement with previous studies (27, 28). The reason for this is that lymph node metastasis implies a more advanced clinical stage and peritumoral edema may be associated with embolic obstruction of the tumor (28); secondly, the marginal enhancement sign represents that the peritumoral area of the tumor (as compared to the central area) exhibits higher glucose uptake and NADH levels (29) or higher levels of lactate and pyruvate (30) and the central region shows a more oxidized metabolic state (29). In addition, we note that there is more overlap between the central region of the tumor and habitat1 in this study, suggesting that there may be a central region of the tumor that corresponds to a more invasive region. This view of ours is corroborated by an ultrasound habitat study (31), whereby the habitat sub-region in the center of the tumor was present as a potential infiltration or a solid region with higher tumor cell density, whereas the tumor margins appeared to be the habitat sub-region containing local fibrosis and calcification.

This study has several limitations. First, this is a retrospective, single-institution study, so there may be selection bias in our patients. Second, our follow-up period was relatively short, and we will further extend the follow-up period in the future. Third, manual outlining of ROIs inevitably has inter-observer variation. Finally, a limitation that exists with most other habitat and radiomics studies is the limitation of histopathology (32), and further histopathological confirmation is still needed for this study.

## Conclusion

In summary, our study demonstrates that higher habitat ITH (>0.58) is associated with worse DFS, and further elucidates the biological implications of the habitat subregions derived from subtraction maps. However, this threshold was derived from a single-institution cohort and requires multi-center external validation before future clinical application. These findings suggest that DCE subtraction mapping serves as a biomarker carrying biological information, offering greater biological interpretability than habitat subregions derived solely from data-driven feature extraction.

## Data Availability

The original contributions presented in the study are included in the article/supplementary material. Further inquiries can be directed to the corresponding author.
